# Identification and Biochemical Characterization of High Mobility Group Protein 20A as a Novel Ca^2+^/S100A6 Target

**DOI:** 10.3390/biom11040510

**Published:** 2021-03-30

**Authors:** Maho Yamamoto, Rina Kondo, Haruka Hozumi, Seita Doi, Miwako Denda, Masaki Magari, Naoki Kanayama, Naoya Hatano, Ryo Morishita, Hiroshi Tokumitsu

**Affiliations:** 1 Applied Cell Biology, Graduate School of Interdisciplinary Science and Engineering in Health Systems, Okayama University, Okayama 700-8530, Japan; p11b7txy@s.okayama-u.ac.jp (M.Y.); pz0925wi@s.okayama-u.ac.jp (R.K.); p7bw0ijk@s.okayama-u.ac.jp (S.D.); magari-m@cc.okayama-u.ac.jp (M.M.); nkanayam@cc.okayama-u.ac.jp (N.K.); nhatano@okayama-u.ac.jp (N.H.); 2 Department of Applied Chemistry and Biotechnology, Faculty of Engineering, Okayama University, Okayama 700-8530, Japan; p53p23r5@s.okayama-u.ac.jp; 3 Cell Free Sciences Co., Ltd., Matsuyama 790-8577, Japan; m-denda@cfsciences.com (M.D.); rmorishita@cfsciences.com (R.M.)

**Keywords:** S100 protein, HMG20A, protein-protein interaction, Ca^2+^-signal transduction, genome-wide screening

## Abstract

During screening of protein-protein interactions, using human protein arrays carrying 19,676 recombinant glutathione s-transferase (GST)-fused human proteins, we identified the high-mobility protein group 20A (HMG20A) as a novel S100A6 binding partner. We confirmed the Ca^2+^-dependent interaction of HMG20A with S100A6 by the protein array method, biotinylated S100A6 overlay, and GST-pulldown assay in vitro and in transfected COS-7 cells. Co-immunoprecipitation of S100A6 with HMG20A from HeLa cells in a Ca^2+^-dependent manner revealed the physiological relevance of the S100A6/HMG20A interaction. In addition, HMG20A has the ability to interact with S100A1, S100A2, and S100B in a Ca^2+^-dependent manner, but not with S100A4, A11, A12, and calmodulin. S100A6 binding experiments using various HMG20A mutants revealed that Ca^2+^/S100A6 interacts with the C-terminal region (residues 311–342) of HMG20A with stoichiometric binding (HMG20A:S100A6 dimer = 1:1). This was confirmed by the fact that a GST-HMG20A mutant lacking the S100A6 binding region (residues 311–347, HMG20A-ΔC) failed to interact with endogenous S100A6 in transfected COS-7 cells, unlike wild-type HMG20A. Taken together, these results identify, for the first time, HMG20A as a target of Ca^2+^/S100 proteins, and may suggest a novel linkage between Ca^2+^/S100 protein signaling and HMG20A function, including in the regulation of neural differentiation.

## 1. Introduction

S100 proteins are members of the two EF-hand calcium-binding protein families, which are composed of 20 paralogs in humans [1]. The physiological functions of S100 proteins are thought to be mediated by interactions with target proteins, thereby regulating their intracellular and extracellular activities [2]. S100A6 (also known as calcyclin), a member of the S100 protein family [3], was first discovered as a growth-regulated gene (2A9) in 1986 [4]. S100A6 mRNA levels are increased by treatment with various growth factors, including serum, platelet-derived growth factor, and epidermal growth factor in quiescent fibroblasts. Since its discovery, biochemical approaches have been used to identify a number of intracellular target proteins for S100A6, including glyceraldehyde-3-phosphate dehydrogenase, annexin II, and VI [5], annexin XI (calcyclin-associated protein 50kDa (CAP-50)) [6], caldesmon [7], tropomyosin [8], p30, CacyBP/SIP (calcyclin-binding protein and siah-1-interacting protein) [9,10], Hsp70/Hsp90-organizing protein, kinesin light chain, translocase of outer mitochondrial membrane 70 [11], protein phosphatase 5 (PP5) [12], FK506-binding protein 38 [13], and C-terminus of Hsc70-interacting protein [14], consistent with its primarily cytoplasmic location. Recently, a tricopeptide repeat (TPR) domain was identified as a common feature of the Ca^2+^-dependent S100A6-binding regions of PP5 [12], Hsp70/Hsp90-organizing protein, kinesin light chain, and translocase of outer mitochondrial membrane 70 [11]. In addition, the extracellular localization of S100A6 was also shown to be important for modulating the viability of the neuroblastoma cell line SH-SY5Y via interactions with the receptor for advanced glycation end-products (RAGE) [15]. The identification of a growing number of S100A6 interactants as described above suggests the multifunctional roles of S100A6, which may be involved in regulating a wide variety of Ca^2+^-signal transduction in the cells and under pathological conditions, including neurodegenerative disorders [16].

To comprehensively analyze target proteins of EF-hand Ca^2+^-binding proteins, including calmodulin and S100 proteins, we developed a novel genome-wide screening method based on the protein–protein interactions using the Protein Active Array^®^ (CellFree Sciences, Ehime, Japan), carrying 19,676 recombinant glutathione s-transferase (GST)-fusion proteins [17]. The use of this method resulted in the identification of the striated muscle activator of Rho signaling as a calmodulin interactant, and FOR-20 (FOP-related protein of 20 kDa) as a novel S100A6-interacting protein [18]. In this study, during the course of human protein array screening with biotinylated S100A6 as a ligand, we discovered HMG20A (high-mobility group protein 20A) as a novel S100A6-binding protein. We further characterized the biochemical interaction between S100A6 and HMG20A in vitro and in living cells.

## 2. Materials and Methods

### 2.1. Materials

S100 proteins (S100A1, A2, A4, A6, A11, A12, and B) were expressed in *Escherichia coli* BL21 (DE3) using the pET vector and purified by phenyl-sepharose chromatography, as described previously [11]. Recombinant S100A6 was biotinylated with biotinoyl-ε-aminocaproic acid N-hydroxysuccinimide ester, followed by purification [19]. Recombinant rat calmodulin was expressed in *E. coli* BL21 (DE3) using the plasmid pET-calmodulin (kindly provided by Dr. Nobuhiro Hayashi, Tokyo Institute of Technology, Yokohama, Japan) [20]. Human HMG20A cDNA in pcDNA3.1/V5-His (Invitrogen, Waltham, MA, USA) [21] and human HMG20B cDNA in pET21a (Merck Biosciences, Darmstadt, Germany) [22] were kind gifts from Dr. Wen Chang (Academia Sinica, Taiwan) and Dr. Ashok Venkitaraman (University of Cambridge, Cambridge, UK), respectively. Anti-GST and anti-His tag antibodies were obtained from GE Healthcare UK (Little Chalfont, UK) and Sigma-Aldrich (St. Louis, MO, USA), respectively. Antibodies against S100A6 and human HMG20A were purchased from Epitomics (Burlingame, CA, USA) and Sigma-Aldrich (St. Louis, MO, USA), respectively. All other chemicals were obtained from standard commercial sources.

### 2.2. Screening of S100A6 Targets Using Human Protein Array

Protein array plates (Protein Active Array^®^, 1536-well format × 27 plates) containing 19,676 recombinant human proteins in duplicate, generated by wheat germ cell-free protein synthesis, were constructed by CellFree Sciences [17], and the screening for protein–protein interactions was performed by incubation with 0.36 µg/mL biotinylated S100A6, followed by a streptavidin–horseradish peroxidase-mediated enhanced chemiluminescence (ECL) detection of S100A6 binding with the ChemiDoc™ XRS (Bio-Rad Laboratories, Hercules, CA, USA), as previously reported [18].

### 2.3. Construction, Expression, and Purification of GST-HMG20A and Mutants

Human HMG20A cDNA was amplified by PCR using PrimeSTAR HS DNA polymerase (Takara Bio, Shiga, Japan) and pcDNA3.1/HMG20A-V5-His, as described above, as a template with a sense primer (5′-gggtctagaaaacttgatgactagctc-3′) and an antisense primer (5′-acgatcgagtctgttcacaacttc-3′ for GST-HMG20A-His_6_). The cDNA was then subcloned into the pGEX-PreS-His_6_ vector (*XbaI/SmaI* site) [23], resulting in pGEX-PreS-HMG20A wild-type (2–347)-His_6_, which was subsequently sequenced. Truncation and deletion mutants of GST-HMG20A-His_6_ were constructed by PCR using PrimeSTAR HS DNA polymerase and the following primer pairs:

GST-HMG20A(2–219)-His_6_: 5′gggtctagaaaacttgatgactagctc3′/5′agggatgtcaaaaacagaccgttcc3′. GST-HMG20A(220–347)-His_6_: 5′gggtctagaaatatttacagaggaattcttg3′/5′acgatcgagtctgttcacaacttc3′. GST-HMG20A(296–347)-His_6_: 5′gggtctagaatttgccagcatgcccttgcc3′/5′acgatcgagtctgttcacaacttc3′. GST-HMG20A(311–347)-His_6_: 5′gggtctagaagacaccattgactc3′/5′acgatcgagtctgttcacaacttc3′. GST-HMG20A(316–347)-His_6_: 5′gggtctagaatatatgaacagactgcacag3′/5′acgatcgagtctgttcacaacttc3′. GST-HMG20A(296–342)-His_6_: 5′gggtctagaatttgccagcatgcccttgcc3′/5′cacaacttctcgaactgtagctatg3′. GST-HMG20A(296–337)-His_6_: 5′gggtctagaatttgccagcatgcccttgcc3′/5′tgtagctatgaagttttcattgtcttg3′.

Mammalian expression plasmids for GST-HMG20A (pME-GST-HMG20A wild-type (WT) and a 2–310 mutant (ΔC)) were constructed by PCR as described above, followed by subcloning into the pME18s vector. The nucleotide sequences of all constructs were confirmed by sequencing using an ABI PRISM 310 genetic analyzer (Applied Biosystems, Foster City, CA, USA). GST-HMG20A-His_6_ proteins, including various mutants, were expressed in *E. coli* BL21 Star (DE3), followed by extraction for S100A6 overlay, or were further purified by glutathione-sepharose chromatography and subsequent Ni-NTA (nickel-nitrilotriacetic acid) chromatography (Qiagen, Hilden, Germany).

### 2.4. GST Precipitation Assay

Recombinant GST-HMG20A-His_6_ (28–45 µg) was incubated with the indicated amounts of S100 proteins in a solution (500 µL) containing 150 mM NaCl, 20 mM Tris-HCl (pH 7.5), and 0.05% Tween 20 in the presence of either 2 mM CaCl_2_ or 2 mM ethylene glycol-bis (β-aminoethyl ether)-N,N,N′,N′-tetraacetic acid (EGTA) for 1 h at room temperature. Subsequently, 50 µL of glutathione-sepharose beads (50% slurry) were incubated with the sample, followed by incubation for 1 h. After extensive washing, 50 µL of 1× SDS-PAGE sample buffer was added to the beads, followed by heating at 95 °C for 10 min. Samples (10 µL) were analyzed on a Tris-Tricine-SDS-10% polyacrylamide gel using either Coomassie Brilliant Blue staining or immunoblot analysis [19].

### 2.5. Cell Culture, Transfection, and GST Precipitation Assay

COS-7 and HeLa cells were cultured in Dulbecco’s modified Eagle’s medium supplemented with 10% fetal bovine serum (FBS), 100 U/mL penicillin, and 100 U/mL streptomycin at 37 °C in 5% CO_2_. COS-7 cells were plated on 10 cm dishes and transfected with 10 µg of an expression plasmid for GST (pME-GST), GST-HMG20A (pME-GST-HMG20A WT), or a 2–310 mutant (pME-GST-HMG20A ΔC) using polyethyleneimine “MAX” (Polysciences, Inc., Warrington, PA, USA), in accordance with the manufacturer’s protocol. After 48 h of culture, the cells were lysed with 1 mL of ice-cold lysis buffer (150 mM NaCl, 20 mM Tris-HCl, pH 7.5, 0.05% Tween20, 0.2 mM phenylmethylsulfonyl fluoride (PMSF), and either 2 mM EGTA or 2 mM CaCl_2_) and centrifuged at 17,970× *g* for 10 min to obtain the cell lysate. The GST precipitation assay was then performed as described above.

### 2.6. Immunoprecipitation

HeLa cells (10 cm dish × 8 plates) were lysed with 4 mL of 150 mM NaCl, 20 mM Tris-HCl pH7.5, 0.2 mM PMSF, and 10 µg/mL trypsin inhibitor and leupeptin, and 1 mL cell extracts were pre-cleared with 50 µL of Protein G-Sepharose (50% slurry) at 4 °C for 1 h. The pre-cleared cell lysate was incubated with either normal rabbit IgG (1 µg) or anti-HMG20A antibody (1 µg) at 4 °C for 1 h in the presence of either 2 mM CaCl_2_ or 2 mM EGTA. After the addition of 50 µL of Protein G-Sepharose (50% slurry), the samples were further incubated at 4 °C overnight with gentle end-over-end mixing. Immuno-complexes were washed with 1 mL of wash buffer (150 mM NaCl, 20 mM Tris-HCl, pH 7.5, 0.05% Tween20, 0.2 mM PMSF, and either 2 mM EGTA or 2 mM CaCl_2_) five times, followed by the addition of 50 µL of 1× SDS-PAGE sample buffer and boiling at 98 °C for 10 min. Subsequently, the samples were subjected to immunoblot analysis.

### 2.7. Other Methods

Immunoblot analysis was performed using the indicated primary antibodies and with horseradish peroxidase-conjugated anti-mouse, anti-rabbit IgG (GE Healthcare UK, Ltd., Little Chalfont, UK), or anti-goat IgG (Southern Biotech, Birmingham, AL, USA) as the secondary antibody. An ECL reagent (Perkin Elmer, Inc., Waltham, MA, USA) was used for signal detection. Quantification of the amount of protein on the SDS-PAGE gel was performed by using the ImageJ software [24]. The S100A6 overlay method, using biotinylated S100A6 (0.36 µg/mL) in the presence of either 2 mM CaCl_2_ or 2 mM EGTA, was performed as previously described for the calmodulin overlay [25]. Protein concentrations were estimated by staining with Coomassie Brilliant Blue (Bio-Rad Laboratories, Hercules, CA, USA), using bovine serum albumin as a standard.

## 3. Results

### 3.1. Identification of HMG20A as an S100A6 Interacting Protein

**Figure 1:**
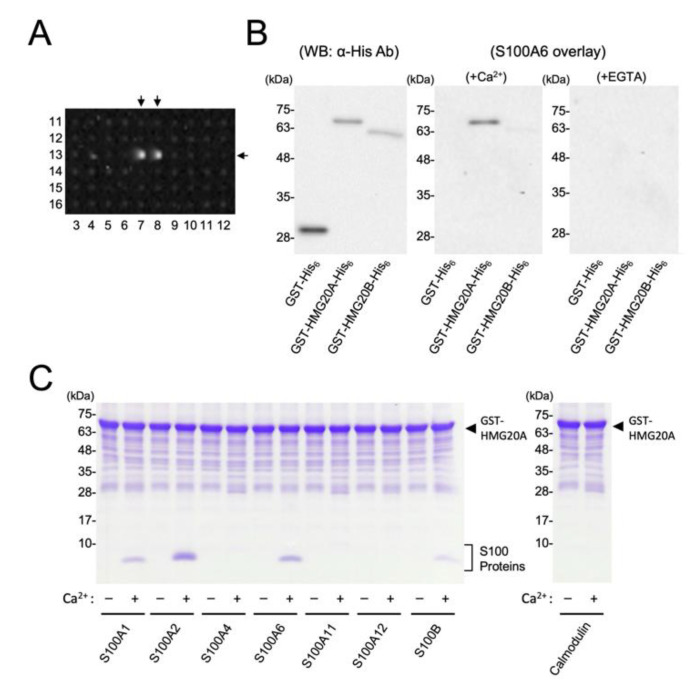
Identification of HMG20A as an S100A6-binding protein. (**A**) A human protein array plate assayed for Ca^2+^/S100A6 binding. Ca^2+^/biotinylated S100A6 binding signals of GST-HMG20A (13:7,8) are indicated by arrows (see Supplemental Figure S1). (**B**) Purified GST-His_6_, GST-HMG20A-His_6_, and GST-HMG20B-His_6_ (0.1 µg) were separated by SDS-10% PAGE, followed by immunoblot analysis using anti-His-tag antibody (left panel), and an S100A6-overlay analysis in the presence of either 2 mM CaCl_2_ (middle panel) or 2 mM EGTA (right panel). (**C**) GST-HMG20A-His_6_ (45 µg) was incubated with S100 family proteins (15 µg), including S100A1, S100A2, S100A4, S100A6, S100A11, S100A12, S100B, and calmodulin (CaM), and then precipitated with glutathione-sepharose beads in the presence (+) or absence (−) of CaCl_2_, as described in Section 2.4. The precipitated samples were analyzed by Tris-Tricine-SDS-10% PAGE, followed by Coomassie Brilliant Blue staining. Arrow heads indicate GST-HMG20A-His_6_. The left-hand lanes in panels B and C show the molecular mass markers.

During the screening, with a protein array system containing 19,676 human full-length cDNA-derived recombinant GST-fusion proteins with biotinylated S100A6 in the presence of 2 mM CaCl_2_ [18], we detected a relatively high-intensity binding signal, pair 13:7,8 (Figure 1A) in the No. 6 array plate, which contains 739 recombinant human proteins in duplicate (Supplemental Figure S1). It was identified as a high-mobility group (HMG) domain-containing protein (HMG20A/iBRAF), which is a non-histone DNA binding protein [26,27].

We therefore decided to further analyze the interaction of HMG20A with S100A6 protein. By using the biotinylated S100A6 overlay, we confirmed that recombinant GST-HMG20A-His_6_ was capable of interacting with S100A6 in a Ca^2+^-dependent manner, but not GST-His_6_ protein, on the blotted membrane subsequently subjected to SDS-PAGE (Figure 1B). Notably, S100A6 did not interact, or interacted only very weakly, with the close homolog of HMG20A, HMG20B/BRAF35 [26] fused with GST, and His_6_-tag in the presence of Ca^2+^. We further analyzed the interaction of HMG20A and various S100 family proteins, including S100A6 under native conditions by using a GST-pulldown assay (Figure 1C). In vitro, GST-HMG20A is capable of interacting with S100A6 only in the presence of 2 mM CaCl_2_, but not GST control (data not shown). Since the S100 family consists of 20 two-EF-hand proteins [1], we attempted to test the binding specificity of HMG20A with six other recombinant S100 proteins—namely, S100A1, A2, A4, A11, A12, B, and calmodulin—using the GST-pulldown assay in the presence or absence of 2 mM CaCl_2_ (Figure 1C). This binding assay clearly showed that S100A1, A2, and B are capable of interacting with HMG20A in a Ca^2+^-dependent manner, but not with A4, A11, A12, and calmodulin. Taken together, this work demonstrates, for the first time, that HMG20A is a novel Ca^2+^/S100-binding protein.

### 3.2. Identification of S100A6-Binding Domain in HMG20A

**Figure 2:**
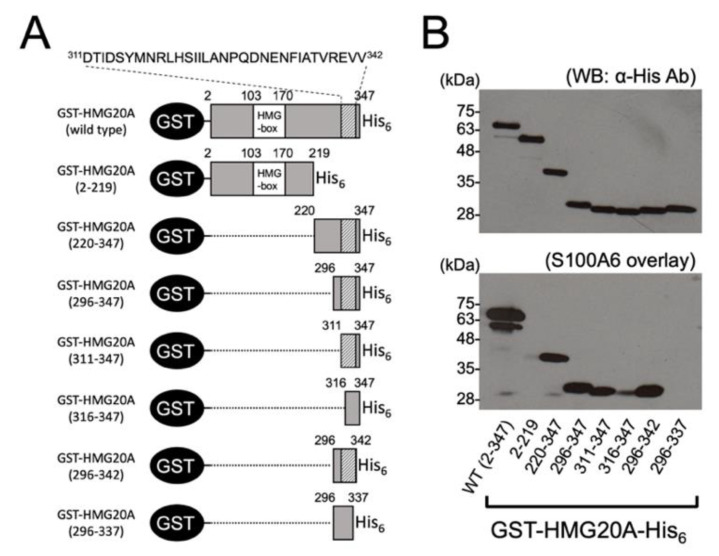
Mapping of the S100A6 binding region in HMG20A. (**A**) Schematic representation of GST-HMG20A-His_6_ proteins, including the wild type (residues 2–347) and various truncation mutants. S100A6 binding sequence (residues 311–342) is indicated. HMG-box, high mobility group-box. (**B**) Extracts of *E. coli* expressing GST-HMG20A-His_6_ proteins as shown in panel A were subjected to immunoblot analysis using anti-His-tag antibody (upper panel) and S100A6 overlay analysis (lower panel).

To map the S100A6-binding region in HMG20A, we expressed a series of N-terminal and C-terminal truncation mutants of GST-HMG20A-His_6_ proteins (2–219, 220–347, 296–347, 311–347, 316–347, 296–342, 296–337) (Figure 2A), and tested their Ca^2+^/S100A6-binding abilities by S100A6 overlay assay (Figure 2B). Equal amounts of the GST-HMG20A-His_6_ mutants, including the wild type loaded on the blotted membrane, were confirmed by immunoblot analysis using anti-His tag antibody (Figure 2B, upper panel), and assayed by S100A6 overlay in the presence of 2 mM CaCl_2_ (Figure 2B, lower panel). While wild-type GST-HMG20A-His_6_ could bind S100A6, deletion of the 128 C-terminal residues (GST-HMG20A 2–219) completely eliminated the binding of S100A6, consistent with the fact that the C-terminal fragment with residues 220–347 was shown to bind Ca^2+^/S100A6.

Although two other C-terminal fragments with residues 296–347 and 311–347 are capable of binding with S100A6 in a manner comparable to that of GST-HMG20A 220–347, the further deletion of five residues (GST-HMG20A 316–347) significantly weakened the S100A6-binding ability, indicating that the S100A6-binding region is located at the C-terminal end of HMG20A. The deletion of five residues from the C-terminal end (GST-HMG20A 296–342) did not affect the S100A6-binding ability, but the further deletion of an additional five residues (GST-HMG20A 296–337) resulted in the complete loss of its S100A6-binding. Residues 338–342 in HMG20A, in particular, seem to be essential for efficient binding to S100A6.

### 3.3. Stoichiometric Binding of S100A6 with HMG20A

**Figure 3:**
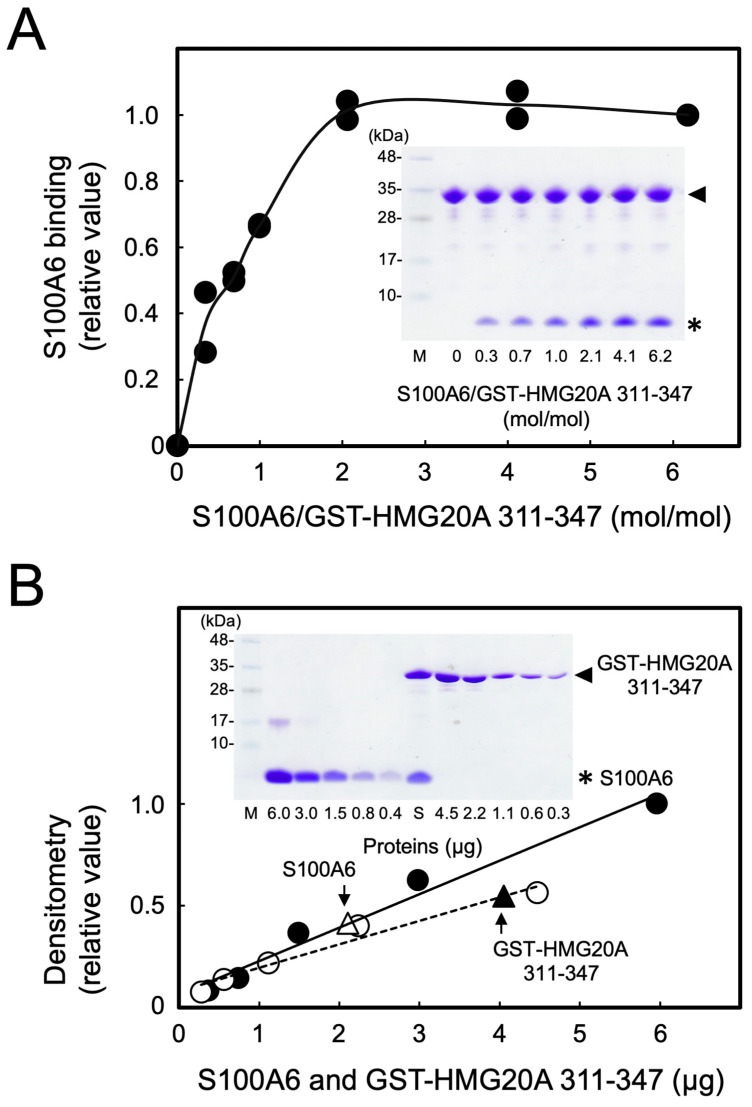
Stoichiometry of S100A6 and HMG20A binding. (**A**) GST-HMG20A 311–347-His_6_ (28 µg) was incubated with indicated amounts of 100A6 (0–54 µg), and then precipitated with glutathione-sepharose beads in the presence of 2 mM CaCl_2_. The precipitated samples were analyzed by Tris-Tricine-SDS-10% PAGE, followed by Coomassie Brilliant Blue staining (insert), and then S100A6 binding was quantified by densitometric scanning as described in Section 2.4. S100A6 binding to GST-HMG20A 311–347-His_6_ was quantified, expressed as a relative value of the sample (S100A6/GST-HMG20A 311–347-His_6_ = 6.2), and plotted from duplicate experiments. (**B**) A pulldown sample (S100A6/GST-HMG20A 311–347-His_6_ = 4.1 in panel A) was analyzed by Tris-Tricine-SDS-10% PAGE together with various amounts of S100A6 (closed circle) and GST-HMG20A 311–347-His_6_ (open circle) as standards, respectively, followed by Coomassie Brilliant Blue staining (insert) to calculate the stoichiometry of S100A6/GST-HMG20A 311–347-His_6_ binding. Arrows indicate the amounts of S100A6 and GST-HMG20A 311–347-His_6_, respectively, in the pulldown sample (lane S in insert). Arrowheads and asterisks in the inserts indicate GST-HMG20A 311–347-His_6_ and S100A6, respectively. Molecular masses of S100A6 and GST-HMG20A 311–347-His_6_ were calculated to be 10,180 and 32,734, respectively. The left-hand lanes (M) in the insert of panels A and B show the molecular mass markers.

To examine the binding stoichiometry of S100A6 with HMG20A, a GST-pulldown assay using GST-HMG20A 311–347, which was shown to bind S100A6 (Figure 2B), was performed with various concentrations of S100A6 (Figure 3A). S100A6 binding to GST-HMG20A 311–347 increased with the addition of an increasing concentration of S100A6, which was saturated at a 1:2 molar ratio (GST-HMG20A:S100A6 monomer). We next attempted to quantify S100A6 and GST-HMG20A 311–347 in the GST-pulldown sample at a 1:4.1 molar ratio, as shown in Figure 3A, based on the densitometric scanning with standard proteins (Figure 3B). In an aliquot of the GST-pulldown sample, 4.1 µg of GST-HMG20A 311–347 and 2.1 µg of S100A6 were calculated to be contained, indicating that the molar ratio of GST-HMG20A 311–347 to the bound S100A6 monomer was 1 to 1.65. Taken together (Figure 3A,B), the S100A6 dimer binds to the HMG20A monomer stoichiometrically at the C-terminal region (residues 311 to 347).

### 3.4. Interaction of HMG20A with Ca^2+^/S100A6 in Cultured Cells

**Figure 4:**
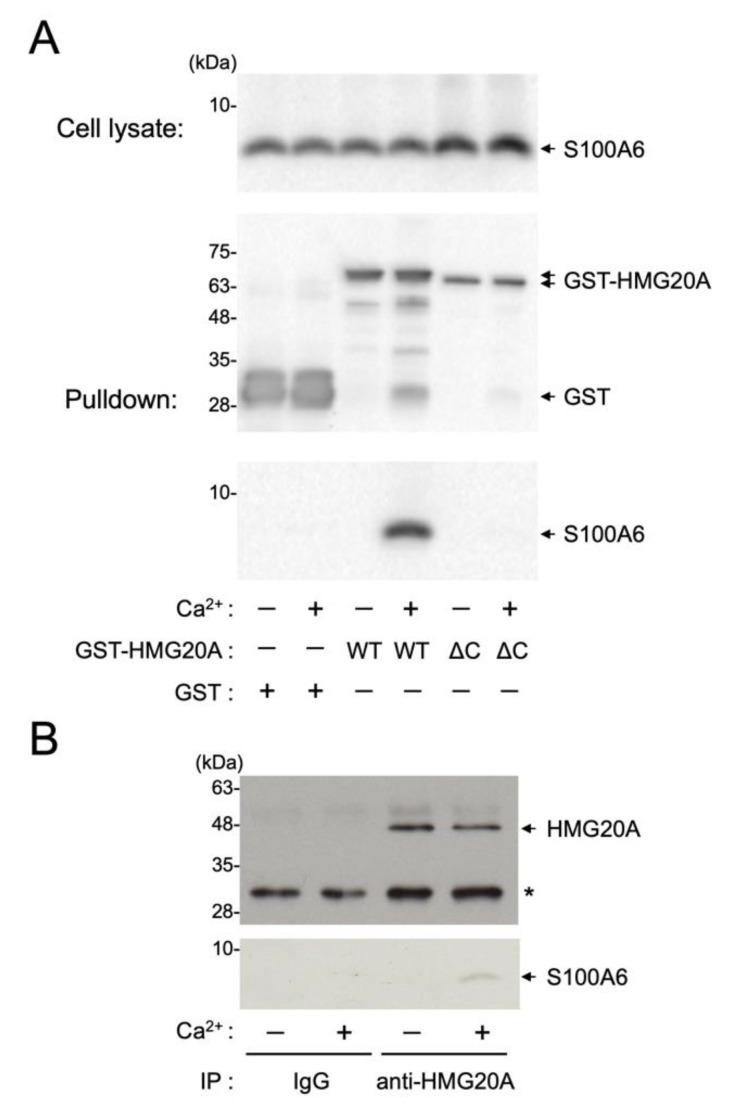
Interaction of HMG20A and S100A6 in cultured cells. (**A**) COS-7 cells were transfected with expression vectors encoding GST, wild-type GST-HMG20A (WT), or its C-terminal deletion mutant (residues 2–310, ΔC). After 48 h of culture, GST-fusion proteins were precipitated with glutathione-sepharose in the presence (+) or absence (−) of CaCl_2_, and the precipitates were subjected to immunoblot analysis using either an anti-GST antibody (middle panel) or an anti-S100A6 antibody (bottom panel) as described in Section 2.5. The cell lysates (10 µL) from the transfected COS-7 cells were analyzed by immunoblotting with an anti-S100A6 antibody (upper panel). (**B**) HeLa cells were immunoprecipitated with either normal rabbit IgG or anti-HMG20A antibody in the presence (+) or absence (−) of CaCl_2_, followed by immunoblot analysis using anti-HMG20A antibody (upper panel) or anti-S100A6 antibody (lower panel), as described in Section 2.6. The left-hand lanes in panels A and B show the molecular mass markers.

We next examined whether the S100A6–HMG20A interaction occurs in intact cells (Figure 4A). COS-7 cells were transfected with either an expression plasmid encoding wild-type GST-HMG20A or a C-terminal truncation mutant (2–310, ΔC) lacking the S100A6-binding region (residues 311–342, Figure 2). GST-HMG20A proteins were then pulled down with glutathione-sepharose in the absence or presence of Ca^2+^ (Figure 4A middle panel). Immunoblot analysis of the precipitated samples with an anti-S100A6 antibody revealed that endogenous S100A6 (Figure 4A, upper panel) in COS-7 cells was co-precipitated with only wild-type GST-HMG20A in the presence of CaCl_2_, not with either the HMG20A-ΔC mutant or GST alone (Figure 4A, lower panel). This clearly indicates that S100A6 is capable of interacting with HMG20A in a Ca^2+^-dependent manner via its C-terminal region in transfected cells. To further characterize the physiological relevance of the S100A6/HMG20A interaction, immunoprecipitation from HeLa cell extracts with anti-HMG20A antibody in the presence of either 2 mM CaCl_2_ or 2 mM EGTA was performed. As shown in Figure 4B, endogenous S100A6 was co-immunoprecipitated with HMG20A from HeLa cell extracts only in the presence of Ca^2+^, indicating the in vivo interaction of Ca^2+^/S100A6 and HMG20A.

## 4. Discussion

In this study, we identified HMG20A as a novel S100A6-binding protein by screening human recombinant protein arrays with a biotinylated S100A6 probe. This interactome approach, with high throughput and high sensitivity, was recently used to identify FOR20 as a novel S100A6-binding protein [18]. HMG20A is known as an inhibitor of BRAF35 (iBRAF), an HMG-containing protein, and plays an important role in the initiation of neuronal differentiation through the activation of neuron-specific genes [28]. HMG20A has been shown to inhibit the sumoylation of HMG20B (BRAF35) and the interaction of HMG20B with the Lys-specific demethylase 1(LSD1)-REST co-repressor 1 (CoREST) complex through heterodimerization of HMG20A and HMG20B, resulting in neuronal differentiation [29]. It was also demonstrated that HMG20A can replace HMG20B in the LSD1–CoREST complex and is involved in mesenchymal transition [30], although the regulatory mechanism of HMG20A is still unclear. This study is the first demonstration that the interaction of S100A6, as well as other S100 family proteins, with HMG20A occurs in a Ca^2+^-dependent manner in vitro and in living cells, but not with HMG20B. It has been reported that S100A6 is found in both the nucleus and the cytoplasm in many mature epithelia and in all endothelial cells, stromal cells such as fibroblasts and myofibroblasts, and nerve sheath cells [31], and enriched at the nuclear envelope in A431 human epidermoid carcinoma cells [32], consistent with the nuclear localization of HMG20A. Therefore, the interaction of S100A6 with HMG20A possibly occurs in the nucleus. Identification of the S100A6-binding region in HMG20A (residues 311–342) confirmed the specific interaction of the S100 protein with HMG20A; however, it showed no significant homology of S100A6-binding sequences between HMG20A and other S100A6 binding partners, including Siah-1 interacting protein (SIP [33]), FOR-20 [18], Annexin XI-A [34], and human Sgt1 [35] (Table 1). We observed the HMG20A monomer bound with S100A6 dimer (Figure 3), which might occur in a similar manner to the SIP/S100A6 interaction [33]. A crystallographic study of the RAGE/S100A6 complex indicated that two RAGE ectodomains interact with one S100A6 homodimer [36]; therefore, the structural analysis of the HMG20A/S100A6 complex would be necessary to understand the diversity of the S100A6/target interaction. Previously, it was shown that HMG20A is capable of interacting with β-dystrobrevin, a cytoplasmic component of the dystrophin-associated protein complex, resulting in the regulation of chromatin dynamics, possibly playing a role in neuronal differentiation [37]. The interaction of β-dystrobrevin and the C-terminal fragment (residues 233–342) of HMG20A [37], containing the S100A6-binding region (residues 311–342), raises the possibility that S100A6 may regulate the HMG20A/β-dystrobrevin interaction. This is in agreement with the high expression of S100A6 in neural stem cells [38]. Therefore, we will test our hypothesis to determine whether S100 proteins regulate the HMG20A function in vivo in a Ca^2+^-dependent manner, including the regulation of HMG20A/β-dystrobrevin interaction that may control the neuronal gene expression, in a future study.

## Figures and Tables

**Table 1:** S100A6-binding sequences of S100A6 target proteins.

S100A6 Targets		S100A6-Binding Sequence	Reference
human HMG20A	311	DTIDSYMNRLHSIILANPQDNENFIATVREVV 342	this study
mouse SIP	189	SEGLMNVLKKIYEDGDDDMKRTINKAWVESR 219	[33]
human FOR20	1	MATVAELKAVLKDTLEKKGVLGHLKARIRA 30	[18]
rabbit Annexin XI-A	49	QDYLSGMAANMSGT 62	[34]
human Sgt1	263	KEEEKNEKLEGDAALNRLFQQIYSDGSDEVKRAM	[35]
		NKSFMESGGTVLSTNWSDVGKRKVEINPPDDMEW	
		KKY 333	

## Supplementary Materials

The following are available online at https://www.mdpi.com/2218-273X/11/4/510/s1, Figure S1: Identification of HMG20A as an S100A6-binding protein by Protein Active Array^®^ screening.
