# Supplementary material for: Identification and Biochemical Characterization of High Mobility Group Protein 20A as a Novel Ca2+/S100A6 Target
Source: Biomolecules. 2021 Mar 30;11(4):510. doi: 10.3390/biom11040510 (PMC8103281; doi:10.3390/biom11040510)
Supplement: Supplementary file 1 [file biomolecules-11-00510-s001.zip › biomolecules-1137753-supplementary.pdf]

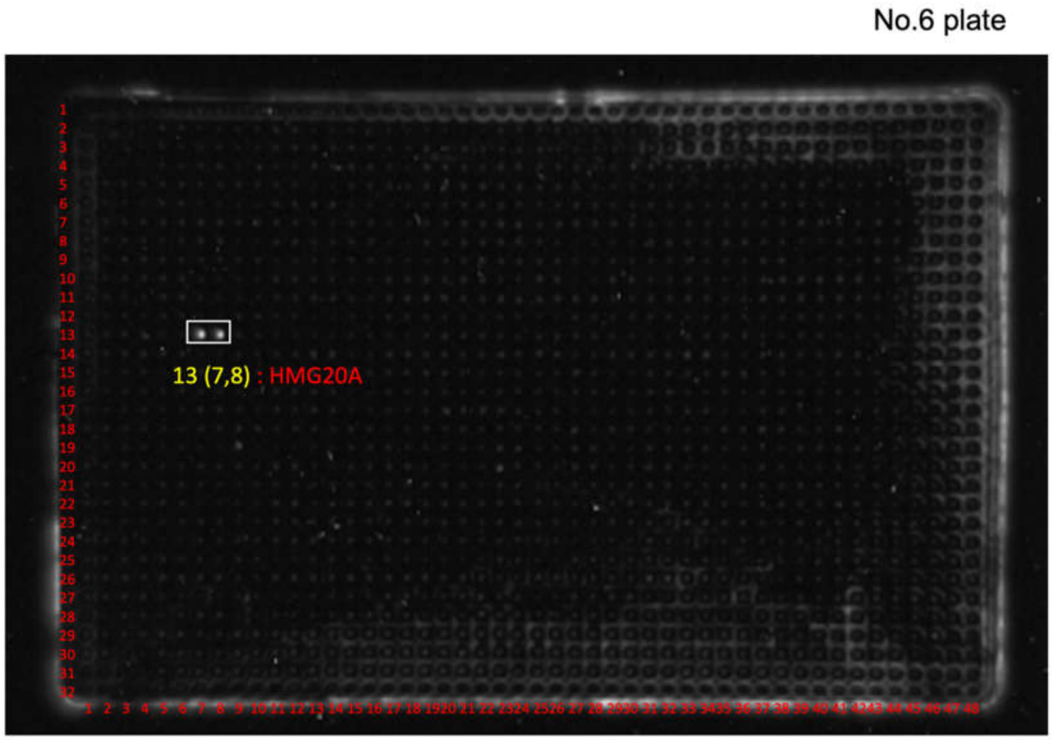

Supplemental Figure. S1. Identification of HMG20A as an S100A6-binding protein by Protein Active Array® screening.

The screening result of biotinylated S100A6 binding on the No. 6 array containing 739 duplicate human proteins (01:05,06–32:43,44) with positive signals at 13:7,8 corresponding to HMG20A (high mobility group protein 20A isoform a [*Homo sapiens*], accession number: NP_060670.1).
